# Non-invasive assessment of free steroid hormones: development of a high-throughput LC–MS/MS method for salivary steroid hormone quantification

**DOI:** 10.1007/s00216-025-06005-9

**Published:** 2025-07-18

**Authors:** Tove Slettvoll, Jeanette Wahlberg, Yvonne Lood, Martin Josefsson, Elisabeth Aardal, Samira Salihovic

**Affiliations:** 1 School of Medical Sciences, Faculty of Medicine and Health, Örebro University, Örebro, Sweden; 2 Department of Endocrinology, Faculty of Medicine and Health, Örebro University, Örebro, Sweden; 3 Division of Clinical Chemistry, Department of Biomedical and Clinical Sciences, Linköping University, Linköping, Sweden; 4 Department of Forensic Genetics and Forensic Toxicology, National Board of Forensic Medicine, Linköping, Sweden; 5 Department of Biomedical and Clinical Sciences, Linköping University, Linköping, Sweden; 6 National Forensic Centre, Linköping, Sweden; 7 Department of Physics, Chemistry and Biology, Linköping University, Linköping, Sweden

**Keywords:** Extraction, Free steroid hormones, Saliva, LC–MS/MS, Unispray ionization

## Abstract

Steroid hormone concentrations reflect diverse physiological and pathological processes and have been recognized as valuable biomarkers for disease, with growing interest in their potential for patient stratification in precision medicine. Salivary steroid concentrations should reflect the free (biologically active) steroids in circulation, as steroids in the bloodstream passively diffuse to saliva. This allows for the direct measurement of free steroids without transporter protein–bound hormones (inactive form). However, implementation of salivary steroid quantification in larger studies remains limited by challenges associated with sample preparation. We began by reviewing the literature on relevant sample preparation approaches and identified commonly used 96-well solid phase extraction (SPE) methods for further evaluation. Three sample preparation methods were selected and assessed in terms of internal standard recovery and matrix effects, and their response was also compared using electrospray (ESI) versus UniSpray ionization (USI) liquid chromatography-tandem mass spectrometry (LC–MS/MS). We demonstrate a sensitive and rapid high-throughput method with Oasis HLB µElution SPE of 200 μL saliva in 96-well format and USI-LC–MS/MS for major steroids (testosterone, androstenedione, cortisone, cortisol, and progesterone) in saliva. The method achieved optimal recovery (77%), matrix effects (33%), and sensitivity with detection limits (MDL) ranging between 1.1 and 3.0 pg/mL and linearity (*r*^2^ = 0.99). Intra-plate and inter-plate coefficient of variation (CV) was below 7% and 20% using USI. USI provided a higher (2.0–2.8-fold) response than ESI and higher signal-to-noise ratio (S/N). The method was then applied to 97 authentic saliva samples (41 male and 56 female) and significant correlations between age and BMI, and androgen levels were observed in both sexes. The proposed 96-well SPE USI-LC–MS/MS method is well-suited for determining steroid hormones in saliva with potential usefulness in large-scale studies and clinical settings.

**Graphical Abstract:**

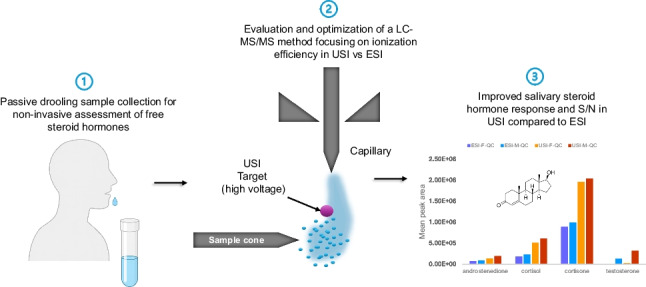

## Introduction

Steroid hormones are essential for the body’s normal function and are, for example, key in human reproduction and immune system regulation (1). Saliva has gained attention in steroid assessment as a less invasive alternative to blood sampling. Moreover, steroids in saliva are usually in their free or unbound state, absent of transporter proteins. This eliminates the need for separation procedures of transporter protein–bound and unbound steroids, which are required in serum free-hormone analysis. Saliva measurement is feasible because several steroids in saliva have been reported to correlate significantly with free steroids in serum (2–5) and should therefore reflect the free, biologically active steroid hormonal levels in the blood, thus providing a non-invasive alternative for steroid hormone assessment (6). However, the accurate quantification of steroid hormones in saliva is challenging due to their complex matrix and low concentrations (7). Another potential challenge is blood or plasma leakage into the oral cavity, which will artificially increase salivary steroid concentrations (8). Enzyme immunoassays can be used for the determination of steroids in clinical settings; however, some immunoassay kits can be prone to cross-reactivity, especially at concentrations as low as in saliva (9) and moreover are not validated for the salivary matrix with detection limits above normal levels (10).

Advances in liquid chromatography-tandem mass spectrometry (LC–MS/MS) have enabled the sensitive and selective analysis of steroid hormones, with the ability to multiplex. The sample preparation is important for extracting the analytes while minimizing interferences. Steroid hormones have been determined by LC–MS/MS in a number of studies, and the most commonly used sample preparation method has been liquid–liquid extraction (LLE) (Table 1). However, to reduce unwanted matrix components and decrease matrix effects, solid-phase extraction (SPE) can be applied, as it reduces interfering components prior to analysis (11) and can be applied in a 96-well format suitable for automation, enabling a high-throughput analysis for large-scale studies.

**Table 1:** Overview of recent analytical methods used for salivary steroid hormone analysis, including methodological parameters and reported concentration range

Participants	Sample preparation		Instrumental analysis	Testosterone conc. (pg/mL) [LOD]	Cortisone conc. (pg/mL) [LOD]	Cortisol conc. (pg/mL) [LOD]	Progesterone conc. (pg/mL) [LOD]	Androstenedione conc. (pg/mL) [LOD]	Ref
	**Sample volume**	**Extraction**							
**Male: 5** **Female: 5**	200	SPE	ESI-UPLC-MS/MS	M: 25–59 F: 2–17 [2]	NA	NA	NA	NA	(16)
**Male: 121**	n.i	RIA	-	M: 53–196 [35]	NA	NA	NA	NA	(19)
**Male: 1145** **Female: 2453**	200	LLE	UPLC-MS/MS	M: 44–91 F: 7–11 [1.9]	NA	NA	NA	NA	(20)
**Children/adolescents: 27**	25	EIA	-	Children: 26–82 [1]	NA	NA	NA	NA	(21)
**Male: 15** **Female: 15**	500	LLE	ESI-UPLC-MS/MS	M: 22–1400 F: 20–140 [6]	M: 540–5000 F: 690–1900 [100]	M: 110–1,542,000 F: 120–1800 [100]	M: 53–440 F: 45–620 [500]		(1)
**Male: 387** **Female:439**	1500	LLE	GC–MS/MS	M: 144–275 F: < LOD [5]	M: 19,200–24,200 F: 18,900–25,500 [250]	M: 7140–13,200 F: 7530–14,200 [150]		M: 117–141 F: 126–173 [10]	(22)
**Male: 50** **Female: 50**	500	LLE	APCI-HPLC–MS/MS	n.i. [0.7]					(3)
**Male: 36** **Female: 47**	100	LLE	ESI-HPLC–MS/MS	M: 18–103 F: < 11 [2.9]				M: 15–100 F: 7–101 [2.9]	(9)
**Male: 60** **Female: 88**	500	LLE	ESI-UPLC-MS/MS	M: 7–99 F: < 1.7–20 [1.7]	M: 50–19,440 F: 540–20,520 [70]	M: 69–8337 F: 76–8337 [11]	M: < 2.5–42 F: < 2.5–55 [2.5]		(7)
**NA**	300	Online SPE	ESI-UPLC-MS/MS	M: 21–99 F: 1.4–13 [n.i.]	Both: 6480–16,920 [n.i.]	Both: 943–16,675 [n.i.]	n.i		(7)
**Male: 1** **Female:1**	500	LLE, derivatization	ESI-HPLC–MS/MS	n.i. [0.3]			n.i. [0.05]	n.i. [0.25]	(23)
**Male: 10** **Female:10**	100	LLE	ID-UPLC-MS/MS	Both: 1.7–87 [0.9]	Both: 6516–25,884 [n.i.]	Both: 471–721 [n.i.]		Both: 21–148 [n.i.]	(24)
***N*: 16 (gender not stated)**	100	Online SPE	APCI-HPLC–MS/MS	14.8–128 [5]	Both: 2170–17,300 [3]	Both: 63.4–854 [5]	Both: 14–48.6 [5]		(25)
**Male: 48** **Female:61**	200	SLE	ESI-UPLC-MS/MS (QTrap)	M: 19.9–29.8 F: 4.5–9.1 [50]	M: 1517–8608 F: 1232–9432 [50]	M: 261–2757 F: 249–2720 [50]	M: 9.3–99.0 F: 3.9–85.6 [1250]	M: 20.0–60.4 F: 4.5–45.9 [500]	(26)
**Female: 9**	50	RIA	-	F: 0–1.47 [n.i.]		F: 430–1970 [20]	F: 0.76–12.74 [2.75]		(27)
**Male: 56** **Female:42**	500 (25–75)*	LLE (EIA)*	LC–MS/MS	M: 63.6 F: 11.6 [1] (M: 127–222 F: 45.0–81.9)* [1–1.86]*					(28)
**Male: 696** **Female: 1352**	100	EIA	-	M: 10.2–26.7 F: 10.3–26.7 [1]					(29)

Note: *ESI* electrospray ionization, *APCI* atmospheric pressure chemical ionization, *UPLC* ultra performance liquid chromatography, *HPLC* high-performance liquid chromatography, *GC* gas chromatography, *MS/MS* tandem mass spectrometry, *RIA* radioimmunoassay, *EIA* enzyme immunoassay, *ID* isotope dilution, *LLE* liquid–liquid extraction, *SPE* solid phase extraction, *SLE* supported liquid extraction, *n.i.* not indicated, *LOD* limit of detection

*EIA and LC–MS/MS are compared; parameters and results for EIA are presented in parentheses. Sample volume and LOQ are obtained from the manufacturers

Additionally, the ionization efficiency is a key factor affecting sensitivity in LC–MS/MS hormone analysis (12, 13) where most studies analyzing salivary steroids have applied electrospray ionization (ESI) (Table 1). Recently, UniSpray ionization (USI), a technique using an impactor rod bending the ions upon collision into the mass spectrometer, has shown potential for improving sensitivity in detecting analytes such as cortisol and cortisone in saliva (14). It is unclear whether USI could improve the peak area and signal-to-noise ratio (S/N) and thereby the limit of quantification of steroid hormones in saliva.

This study aimed to review existing salivary steroid methods, then develop and evaluate different 96-well extraction LC–MS/MS methods for measuring major detectable steroids (testosterone, androstenedione, cortisone, cortisol, and progesterone) in saliva. Three SPE procedures were evaluated for recovery and matrix effects, while the ionization efficiency (ESI vs. USI) was optimized for sensitivity. The optimal method was then applied to authentic saliva samples from both men (*n* = 41) and women (*n* = 56) and to explore the correlation between steroids, age, and body mass index (BMI) comparison to previous studies.

## Materials and methods

### Chemicals and reagents

Testosterone, androstenedione, progesterone, cortisone, cortisol, 17-hydroxyprogesterone, 21-deoxycortisol, 11-deoxycorticosterone, corticosterone, hydrocortisone-d4 (product number; 26,500, ≥ 99%), and progesterone-d9 (product number; 25,047, ≥ 98%) were purchased from Cayman Chemicals (Tallinn, Estonia); internal standard testosterone-2,3,4-^13^C_3_ (product number; T070-1ML, certified reference material grade), androstene-3,17-dione-2,3,4-^13^C_3_ (product number; A-084-1ML, certified reference material grade), cortisone-2,3,4–^13^C_3_ (product number; 803,154−1ML, 98 atom % ^13^C, 97% (CP)), and artificial saliva for pharmaceutical research were purchased from Sigma-Aldrich (Solna, Sweden); and recovery standard testosterone-2,2,4,6,6-d5 (product number TRC-KIT7330-1X1ML) was purchased from LGC Standards (Middlesex, UK). Reagents used for extraction and mobile phases were methanol, acetonitrile, water (all Optima™ HPLC grade with purity greater than 99%), and formic acid (98–100%), purchased from Fisher Scientific UK (Loughborough, UK).

### Saliva samples

The salivary steroid concentration is based on male (*n* = 41) and female (*n* = 56) volunteers sampled at the Department of Endocrinology at the University Hospital, Linköping, Sweden, and at the Department of Endocrinology at the University Hospital, Örebro, Sweden. The sampling of adults and analysis of free steroids with LC–MS/MS was approved by the Swedish Ethical Review Authority, Dnr 2019- 05172 and Dnr 2020–04525.

Saliva was collected in the morning between 08:00 and 10:00 AM by passive drooling using Salimetrics® saliva collection aid. The samples were stored at − 80 °C until analysis to break down mucopolysaccharides (15). The volunteers were instructed to avoid eating, drinking, and brushing teeth at least 1 h before sample collection to prevent blood contamination. Exclusion was made if the participants reported oral inflammation and spontaneous bleeding or if the sample was visually contaminated with blood. Participants filled out a consent form and answered questions that could affect the analytical outcome. In the case report file (CRF), the following information was noted: gender, date of birth, BMI, use of any hormonal preparations and/or tobacco, and details regarding the menstrual cycle.

### Sample preparation

Based on the literature review (Table 1), the majority of studies measuring low concentrations of salivary steroids, particularly testosterone in female participants, used LLE, SPE, and RIA. SPE was reported to provide reduced matrix effects compared to LLE (11). To enable high-throughput processing and compatibility with automated workflows, a 96-well microelution SPE format was selected. Among the most frequently applied SPE sorbents were mixed mode anion exchange (MAX) (16, 17) and hydrophilic lipophilic balanced reversed phase (HLB) (18) which were therefore included for method evaluation. We also evaluated 4% phosphoric acid for protein denaturation but observed no significant differences in response or internal standard recovery compared to formic acid. Since formic acid has greater compatibility with mass spectrometry, we proceeded with formic acid.

An automated pipetting robot Andrew + from Andrew Alliance was used to test three extraction methods (Table 2). Saliva samples were thawed at room temperature and centrifuged for 10 min at 4500 g. The clear supernatant (200 µL) was transferred to clean Eppendorf tubes (1.5 mL) and acidified with 200 µL of 4% formic acid, except in Method 1 where the samples were diluted with 200 µL of methanol and 450 µL of water, followed by the addition of 10 µL of internal standard mixture in order to achieve a final concentration of 200 pg/mL in the LC vial. The mixture was vortexed and centrifuged at 1500 g for 5 min. Before extraction, the SPE plates were conditioned with methanol and water. The three methods evaluated were:

- Method 1: A quaternary amine mixed mode anion exchange Oasis MAX µElution plate 2 mg (Waters Milford, MA, USA) was applied. The plate was loaded with 600 µL sample and washed using 200 µL 1% formic acid (v/v) in 15% acetonitrile and 200 µL of 1% ammonia in 15% acetonitrile as washing solutions and 50 µL of 60% acetonitrile and 50 µL of 30 µM ammonium fluoride as elution solutions.
- Method 2: Oasis MAX µElution plate was loaded with 400 µL sample and washed with 400 µL of 5% methanol. Elution was carried out using 50 µL of methanol, following 50 µL of 2% formic acid in methanol.
- Method 3: Hydrophilic-lipophilic-balanced Oasis HLB µElution Plate 2 mg (Waters Milford, MA, USA) was loaded with 400 µL sample and washed with 400 µL of 5% methanol. Elution was carried out using 2 × 50 µL of methanol.

**Table 2:** An overview of the three 96-well SPE methods that were evaluated for recovery and matrix effects

	**Method**	**Method 1**	**Method 2**	**Method 3**
Sample preparation	**Saliva sample**	200 µL	200 µL	200 µL
	**Dilution solution**	200 µL 4% formic acid in H_2_O	200 µL 4% formic acid in H_2_O	200 µL 4% formic acid or 200 µL 4% phosphoric acid in water
	**Internal standard**	10 µL testosterone-13C	10 µL testosterone-13C	10 µL testosterone-13C
	**µElution SPE cartridge**	OASIS MAX µElution plate 2 mg	OASIS MAX µElution plate 2 mg	OASIS HLB µElution plate 2 mg
	**Protein precipitation**	200 µL MeOH	200 µL of 4% FA	200 µL of 4% FA
	**Conditioning**	300 µL MeOH + 150 µL H_2_O	200 µL MeOH + 200 µL H_2_O	200 µL MeOH + 200 µL H_2_O
	**Wash**	200 µL 1% FA (in 15% ACN) + 200 µL 1% NH_3_ (in 15% ACN)	400 µL of 5% MeOH	400 µL of 5% MeOH
	**Elution**	50 µL 60% ACN + 30 µM NH_4_F	50 µL MeOH + 50 µL 2% FA (in MeOH)	2 × 50 µL MeOH
	**Recovery standard**	10 µL testosterone-d5	10 µL testosterone-d5	10 µL testosterone-d5
Performance evaluation using female QC samples (***n*** = 9)	**% mean IS recovery, (CV%)**	53 (48)	72 (28)	77 (6)
	**% mean matrix effect**	78	36	33

All extracts originating from the three SPE methods were spiked with 10 µL recovery standard, testosterone-d5 (4 ng/mL in MeOH) to enable calculation of internal standard recovery and to monitor instrument performance throughout the analytical run. Extracts from Methods 2 and 3 were diluted with 50 µL of water before instrumental analysis. All three methods were evaluated for recovery and matrix effects. Method 3 was determined to be the optimal sample preparation method and was chosen for further development and evaluation of response in ESI vs. USI.

### Instrumental analysis

Targeted analysis was implemented by applying an ACQUITY PREMIER UHPLC I-Class coupled to a Xevo TQ-XS mass spectrometer operating in positive ESI and USI mode (Waters, Milford, USA). An HSS-T3 C18 column with VanGuard FIT (2.1 × 50 mm, particle size 1.8 µm) was used with an ACQUITY Premier HSS-T3 VanGuard FIT Cartridge (2.1 × 5 mm, particle size 1.8 µm) both from Waters (Milford, MA, USA). The sample compartment preserved the samples at 10 °C. Sample injection volume was 10 µL. Consequently, a 6-s pre- and post-needle wash with methanol/isopropanol/acetonitrile/water (25/25/25/25 v/v/v/v) with 0.1% formic acid was performed. The column temperature was set to 50 °C. Mobile phase A contained 30 µM of ammonium fluoride and B contained methanol. The flow rate was adjusted to 0.300 mL/min with the gradient program: 0 min 50% B; 4.5 min 75% B; 5.5 min 95% B; and 6.5 min 50% B. Multiple reaction monitoring was selected, and the multiple reaction mode (MRM) transitions are provided in Table 3. The following additional settings were used: capillary voltage 2.50 kV, desolvation temperature 350 °C, desolvation gas flow 650 L/h, nebulizer gas flow 7.00 bar, collision gas was argon. Data processing and calculation were made using Masslynx 4.2 software (Waters, Milford, MA, USA).

**Table 3:** Optimized multiple reaction monitoring (MRM) transitions and retention times for the analytes and their corresponding stable isotope labeled internal and recovery standards

Compound	Precursor	Quantification ion	Qualification ion	Cone voltage (eV)	Collision energy 1	Collision energy 2	Retention time
Testosterone	289.3	97	109	40	20	24	3.1
Testosterone-C13	292.3	100.1	112.1	30	25	25	3.1
Androstenedione	287.3	97.1	109.1	40	18	19	2.8
Androstenedione-C13	290.1	100	112.1	40	22	21	2.8
Progesterone	315.2	97	109	40	20	22	4.3
Progesterone-d9	324.3	100	113	40	23	23	4.3
Cortisone	361.2	163.1	121	40	22	28	1.4
Cortisone-C13	364	165.7		40	22		1.4
Cortisol	363.2	121	105	45	22	40	1.6
17-Hydroxyprogesterone	331.2	97	109	40	20	24	3.3
11-Deoxycorticosterone	331.2	97	109	40	20	24	3.0
Corticosterone	347.2	329.3	121	40	14	27	2.2
21-Deoxycortisol	347.2	311	269.1	30	15	15	2.0
Cortisol-d4	367.3	121		45	25		1.6
Recovery standard testosterone-d5	294.2	100.2	113.1	30	22	25	3.1

### Quality assurance and control

#### Linearity

A ten-point matrix-matched calibration curve was prepared using artificial saliva (Sigma-Aldrich, Solna, Sweden) spanning between 0 and 100 ng/mL.

#### Reproducibility

In-house saliva QC (*n* = 18) and (*n* = 1) were used to assess reproducibility and NIST 1950 SRM (Sigma-Aldrich, Solna) to assess accuracy.

#### Carry-over

Carry-over was assessed using a methanol blank after a high-concentrated calibration standard (5 ng/mL).

#### Sensitivity

The method detection limit (MDL) was based on mean + 3SD of procedural blank samples (*n* = 6).

#### Matrix effects

Matrix effects were evaluated and calculated based upon calibration standards, a salivary QC sample spiked with internal and recovery standards before and after extraction.

### Statistical analysis

The steroid concentration and demographic data were log-transformed to approximate a normal distribution prior to analysis. The Pearson pairwise correlation test was then applied to evaluate the strength and direction of the linear relationships between the steroid concentrations, BMI, and age for each sex (Stata 16.1, StataCorp LLC, TX, USA).

## Results

### Evaluation of sample preparation approaches for assessment of salivary steroids

The literature review (Table 1) showed that LLE and SPE were the most commonly used sample preparation methods for quantifying low concentrations of salivary steroids. SPE was suggested to provide less matrix effects as compared to LLE (11). In line with this, we selected a 96-well microelution solid phase extraction format to enable automated, high-throughput processing. Among the available sorbents, mixed mode anion exchange and hydrophilic lipophilic balanced reversed phase were most commonly reported and thus chosen for method evaluation (16–18).

### Evaluation of recovery and matrix effects

The three 96-well SPE protocols (Method 1–Method3) were examined for recovery and matrix effect using pooled female QC saliva samples (*n* = 9). QC samples were spiked with internal standard (4 ng/mL ^13^C_3_-testosterone) prior to and after extraction and analyzed with a calibration standard without matrix for each method, summarized in Table 2. Possible dilution and volume effects were compensated by diluting all the samples to uniform volume: 160 µL, and an aqueous-to-organic percentage ratio of 53–65% organic. The mean peak areas of the QC samples spiked with internal standard before extraction were compared to the QC spiked after extraction. The highest recovery for the internal standard was obtained from Method 3, applying a 2 mg Oasis HLB µElution plate, with a mean recovery of 77 ± 6%. The lowest mean internal standard recovery was observed when using Method 1 applying the Oasis MAX with 53 ± 26%, and Method 2 had a recovery of 72 ± 20%. The matrix effect in saliva was evaluated by dividing the area of the recovery standard (3 ng/mL testosterone-d5) in the extracts by the calibration standard. The results varied between the methods, with ionization suppression of 36% for Method 1, 33% for Method 3, and a higher suppression of 78% for Method 2 (Table 2). The approximate automated extraction time from the thawed saliva sample to the final extract for 96 samples is 2.5 h. Method 3 was further optimized by inclusion of additional stable isotope labeled internal standards; in addition to testosterone-2,3,4-^13^C_3_, we added progesterone-d9, hydrocortisone-d4, androstenedione-2,3,4-13C3, and cortisone-13C3. The recoveries of these internal standards ranged between 53 and 140%. Thus, Method 3 was chosen for extraction of the authentic samples, and the following evaluations are based on that method.

### Linearity and carry-over

The ten-point matrix-matched calibration curve (0, 3, 5, 50, 100, 250, 500, 10,000, 50,000, 100,000 pg/mL) showed an average coefficient of determination (*r*^2^) of > 0.99 and a mean relative response factor (RRF) CV of 6.6% for all analytes. The blank injections showed no signal detection after standard injection (5 ng/mL); hence, carry-over was not observed.

### Reproducibility

The CV of two in-house QC levels (pooled female and male saliva samples) for testosterone, androstenedione, cortisone, and cortisol intra-plate were for pooled female QC: 1.6–3.7%, and pooled male QC: 0.3–6.7%. The inter-plate CV for the pooled female QC was 2.1–20%, and for the pooled male QC, it was 1.2–14% (Table 4). 17-Hydroxyprogesterone, 21-deoxycortisol, 11-deoxycorticosterone, and corticosterone were not detected in the pooled saliva QC and, therefore, excluded from further analysis. In addition, other steroid hormones, such as estradiol, were considered but were either not detectable or consistently below the quantification limits in saliva using our method. The NIST1950 SRM was used as an additional quality control parameter. It contains hormones in frozen human plasma, including total testosterone, and was analyzed in this study to validate the accuracy of the method. The measured concentration for total testosterone was 2.3 ng/mL, and the certified concentration was 2.2 ± 0.05 ng/mL, yielding an accuracy of 95%, suggesting that this method is useful also for serum/plasma samples. Recovery for the authentic samples was established based on the ratio between the internal and instrumental standard, providing an average recovery of 114% (range 93–146%) for 97 samples.

**Table 4:** Measured concentrations and reproducibility of the analytes in pooled female and male salivary QC samples reported in pg/mL

Analyte	Intraday (*N* = 5)		Interday (*N* = 9)	
Mean pg/mL [CV%]	Pooled female saliva QC	Pooled male saliva QC	Pooled female saliva QC	Pooled male saliva QC
Testosterone	16 [2.7]	236 [0.3]	15 [2.7]	224 [7.5]
Androstenedione	158 [1.6]	265 [1.6]	159 [2.1]	264 [1.2]
Cortisone	10,256 [3.6]	13,367 [4.1]	10,294 [11]	12,498 [14]
Cortisol	2412 [3.7]	3843 [6.7]	2503 [20]	3611 [14]

### Sensitivity and selectivity

The method detection limit (MDL) was based on the extracted water blank samples. MDLs of 1.1 pg/mL for testosterone, 3.0 pg/mL for androstenedione, 2.7 pg/mL for 11-deoxycorticosterone, 2.5 pg/mL for progesterone, 2.2 pg/mL for cortisone, and 1.9 pg/mL for cortisol were established.

### USI vs. ESI

**Fig. 1:**
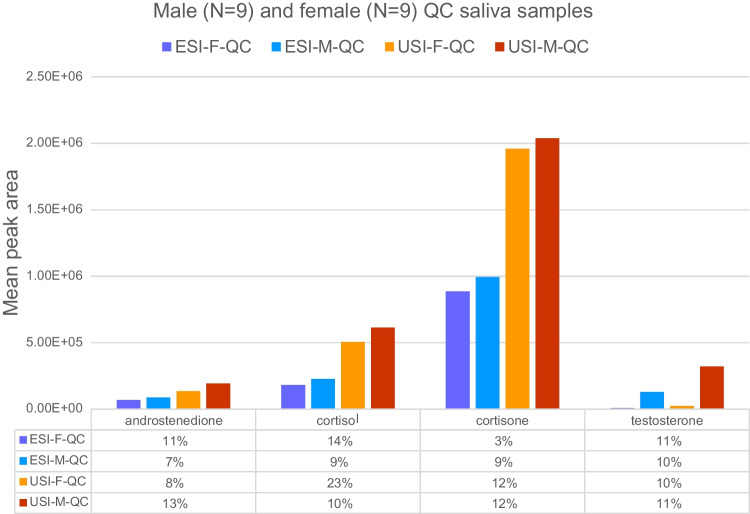
Comparison of electrospray ionization (ESI) and UniSpray ionization (USI) for hormone analysis in QC saliva samples from females and males. Bars represent mean signal areas with standard deviation. USI consistently produced higher signal intensities across all hormones, with approximate fold increases of 2.0–2.2 for androstenedione, 2.7–2.8 for cortisol, 2.1–2.2 for cortisone, and 2.5–2.7 for testosterone

**Fig. 2:**
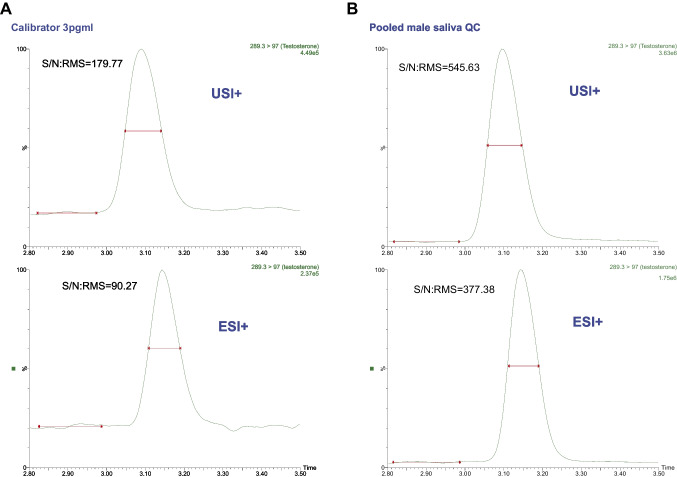
Comparison of testosterone quantifier MRM chromatograms acquired using UniSpray (USI +, top) and electrospray ionization (ESI +, bottom). **A** A matrix-matched calibrator at 3 pg/mL. **B** An extracted pooled male QC saliva sample. Signal-to-noise ratio (S/N) was calculated directly from the raw data using the root mean square (RMS) method

The pooled female and male QC samples (*n* = 18) were analyzed in ESI and USI, and the response evaluated. A 2.0–2.8-fold increase in area was observed for most steroids when using USI as compared to ESI (Fig. 1). In USI, a higher signal-to-noise ratio was observed for a matrix-matched calibration point (Fig. 2A) and for pooled male QC sample (Fig. 2B).

### Application of the method to 97 saliva samples

Steroid median and concentration ranges of male and female participants are presented in Table 5, along with age and BMI. The use of snus and hormone preparation was not included in the statistical analysis because too few participants reported snus use (*n* = 5) or hormonal contraception (*n* = 11). Testosterone and cortisol were detected in all samples, while cortisone was detected in 99%. Androstenedione, detected in 82% of the samples, and progesterone, detected in 43%, showed higher detection frequency in female samples (95% and 52%, respectively) compared to male samples (66% and 32%).

**Table 5:** Overview of the demographic characteristics and salivary steroid concentration (pg/mL) of the study participants

Characteristic	Male	Female	Total
*N*	41	56	97
Age	20–77	23–93	20–93
BMI (kg/m^2^)	18–36	19–49	18–49
Testosterone conc. median [range]	93 [29.5–238]	8.3 [3.5–37.5]	12.5 [3.5–238]
Progesterone conc. median [range]	< 1.25 [< 1.25 to 165.5]	16 [< 1.25 to 582]	< 1.25 [< 1.25 to 582]
Androstenedione conc. median [range]	160 [< 1.5 to 417]	113 [< 1.5 to 474]	126 [< 1.5 to 474]
Cortisone conc. median [range]	16,041 [< 1.2 to 69,716]	13,481 [2828–69,947]	15,270 [< 1.2–69,947]
Cortisol conc. median [range]	3496 [168–85,464]	3152 [169–19,441]	3433 [168–85,464]

**Fig. 3:**
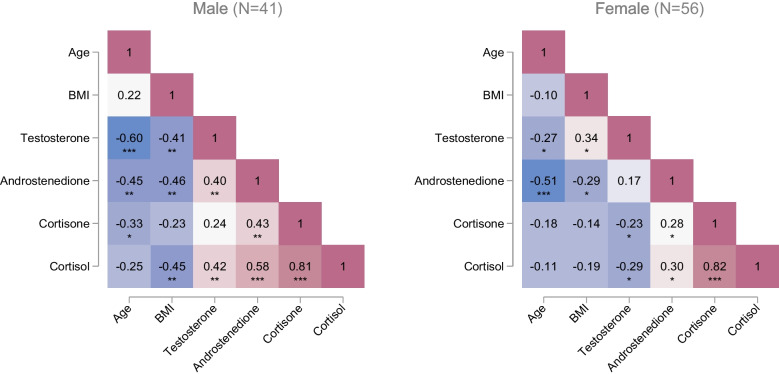
Pairwise correlations between age, BMI, and salivary concentrations of testosterone, androstenedione, cortisone, and cortisol in male (left) and female (right). Note: *p < 0.05, **p < 0.01, and ***p < 0.001

Correlations between the steroid levels, age, and BMI are provided in Fig. 3. Progesterone was excluded due to low detection frequency. Generally, we observed stronger negative correlations between steroids in saliva, age, and BMI among males when compared to females. Steroid levels inversely correlated with age and increased BMI for both sexes, except for female testosterone concentrations where a positive correlation between testosterone and BMI was observed (*r* = 0.34, *p* < 0.05). A significant negative correlation between male testosterone and age was observed (*r* =  − 0.60, *p* < 0.001). Male testosterone significantly correlates with androstenedione and cortisol, while female testosterone shows a negative correlation with cortisone and cortisol. Androstenedione positively correlates with all steroids in both sexes, except with testosterone in females. In both sexes, cortisone and cortisol are strongly correlated (*r* = 0.81, *p* < 0.001).

## Discussion

The main aim of this study was to develop and validate an extraction method for the assessment of salivary steroids. Here, we demonstrate a rapid and high-throughput 96-well-format SPE method using only 200 µL saliva for the determination of steroids as a measure of the free biologically active fraction in plasma that is suitable for larger studies in clinical settings. The method demonstrated a high extraction recovery (93–146%), high sensitivity (MDL 1.1–3.0 pg/mL), and high reproducibility (0.3–20.2%) using the Oasis HLB µElution plate and was possible to automate with a sample throughput of 2.5 h per 96-well plate, thus reducing manual handling.

Previous studies where steroids in saliva were determined most often included LLE. Bakusic et al. [14] evaluated the recovery of salivary cortisol for SPE using an Oasis PRiME HLB cartridge and showed that it provided the highest recovery (98 ± 13%) compared to solid–liquid extraction (9 ± 2%) and LLE (66 ± 25%). In the present study, three different SPE procedures were tested to assess recovery and matrix effects. We found that the 96-well format Oasis HLB µElution plate provided the highest extraction efficiency and satisfactory matrix effects. Method 1 revealed the lowest recovery (53%) which is comparable to the study by Jia et al. [18] where they examined the Oasis HLB µElution plate and the Oasis MAX µElution plate for steroids in human serum and plasma samples and demonstrated higher recovery for the Oasis HLB µElution cartridge (18). The sorbent of an Oasis HLB cartridge consists of two monomers, the hydrophilic N-vinylpyrrolidone and the lipophilic divinylbenzene, while the Oasis MAX was designed for heightened retention of acidic analytes through its strong anion exchange mode (30). Steroids are generally neutral lipid macromolecules, and it could be hypothesized that the higher recovery for the HLB cartridge is due to its lipophilic properties, sorbing the analytes more efficiently. Another possible reason for the higher recovery between Methods 1 and 3 in this study could be the difference in elution solution. Method 3 used 100% organic solution and could have increased the analyte elution compared to the 60% organic solution in Method 1.

The MDLs found in this study are slightly lower (MDL 1.1–3.0 pg/mL vs. 1–100 pg/mL) compared to previous studies analyzing steroids in saliva (1, 7, 9, 25, 28) (Table 1). A possible explanation for lower detection limits could be the ionization technique used. In this study, both ESI and USI were evaluated, and the results showed that the USI provided a 2.0–2.8-fold increase in response compared to the ESI, which has more commonly been used for the analysis of salivary steroids (Table 1). The higher signal-to-noise ratio for testosterone in USI (Fig. 2) further strengthens the conclusion that USI improves detection, particularly for low-abundance analytes. Bakusic et al. [14] demonstrated similar results, where they compared ESI with USI for salivary cortisol and cortisone and demonstrated that USI provided lower detection limits (5 pg/mL vs. 100–500 pg/mL) as well as increased signal intensity. To our knowledge, this is the first time that salivary testosterone and androstenedione have been evaluated in USI.

### Analysis of authentic samples

The optimal method, Method 1 using the Oasis microelution HLB plate, was applied to 97 authentic saliva samples. Testosterone and cortisol were detected in all samples, while cortisone, androsterone, and progesterone had detection frequencies of 99%, 82%, and 43%, respectively. The concentration ranges in the present study are comparable with previous studies using LC–MS/MS to analyze hormones in saliva (7, 9, 20) (Table 1).

We also assessed the pairwise correlations between the salivary steroids and demographic variables including sex, age, and BMI. We found that cortisol and cortisone are strongly correlated in both males and females. These steroids are interconverted by two isomers of 11ß-hydroxysteroid dehydrogenase (11ß-HSD) (31). Notably, the cortisol-to-cortisone ratio differs between the saliva and plasma, with a plasma ratio of approximately 8:1; whereas in the saliva, cortisone concentrations are about 2–6 times higher than cortisol. This inversion is related to the conversion of the unbound cortisol to cortisone via 11ß-hydroxysteroid dehydrogenase 2 (11ß-HSD2) when passing through the salivary gland. A correlation (*r*^2^ = 0.941, *p* < 0.001) between salivary cortisone and cortisol in both genders has also been observed previously (32). Additionally, a positive correlation between salivary cortisol and testosterone was observed in males but not females, which is in line with a previous study (males: *r*^2^ = 0.54–0.73, *p* < 0.01–0.001, females: *r*^2^ =  − 0.12 to 0.22) (33).

The observed negative correlation between testosterone concentration and age is consistent with prior studies where an association between age and decreased total, serum-free, and salivary testosterone concentrations has been consistently reported (20, 34–39). This decline could partially be attributed to the age-related increase in steroid hormone–binding protein (SHBG) levels, which reduces the concentration of free testosterone over time (40). Keevil et al. [20] who examined salivary testosterone in a large cohort study (1145 males and 2453 females aged 18–74) observed a decrease of 1.3–1.5% for men and 1.0–1.4% for women per year with increasing age. Similarly, Kushnir et al. [41] reported declines of androstenedione concentrations with age, where total serum androstenedione levels in males over 40 years decrease by 5% per decade and by 10% per decade in postmenopausal females.

In males, BMI showed a negative correlation between BMI and salivary testosterone, androstenedione, and cortisol, whereas in females, BMI was positively correlated with salivary testosterone. Previous research has identified an inverse correlation between salivary testosterone and BMI in males (42, 43) but not in females (35). Elevated BMI is associated with increased circulating estrogens, most likely caused by the conversion of testosterone to estrogens in adipose tissue (43). Furthermore, higher BMI has been linked to decreased concentrations of SHBG in both sexes (34).

The method sensitivity and multiplexing capacity make it valuable in clinical scenarios such as the evaluation of hypogonadism (45) or follow-up testing in prostate cancer patients receiving anti-androgens (46) (Addison’s disease, congenital adrenal hyperplasia), where hormone concentrations may be subtly altered by chronic disease or physiological stress.

There were some limitations to our analyses. A limitation of the method is that the testosterone concentrations in the study population (*n* = 97) ranged from 3.5 to 238 pg/mL, which is somewhat higher than the concentration of the internal standard (200 pg/mL). We used a matrix-matched calibration approach, and the testosterone showed good agreement with NIST SRM certified values. Despite analyzing 97 samples, expanding the sample size could enable a more comprehensive evaluation of differences in the study population and clarify the potential impact of menstrual cycle, nicotine, and hormonal preparations on salivary hormone levels. Additionally, a larger cohort would improve power to assess associations between BMI, age, and steroids in saliva, particularly in women. Additionally, we did not verify whether the participants complied with the recommended pre-sampling restrictions, such as eating, drinking, and brushing their teeth 1 h prior to sample collection. However, a detailed written protocol was provided to ensure compliance. Furthermore, the presence of blood contamination in saliva samples due to microinjuries was not assessed, such as transferrin analysis, but was instead evaluated through visual inspection.

## Conclusion

To summarize, this study successfully developed a precise and accurate 96-well SPE LC–MS/MS method for quantification of five steroids in saliva. The Oasis HLB 96-well plate 2 mg provided the highest extraction recovery and reduced matrix effects. Furthermore, it is to our knowledge the first study where USI was evaluated for all five steroids simultaneously. USI provided a 2.0–2.8-fold increase in response compared to ESI and an improved signal-to-noise ratio, thereby enhancing ionization and the limit of detection. Significant intercorrelations between steroids in saliva were observed, with age and BMI correlating with androgen levels in both sexes. The proposed 96-well SPE USI-LC–MS/MS method is well-suited for determining steroid hormones in saliva with potential usefulness in clinical settings.

## Data Availability

The datasets generated during and/or analyzed during the current study are available from the corresponding author on reasonable request.
